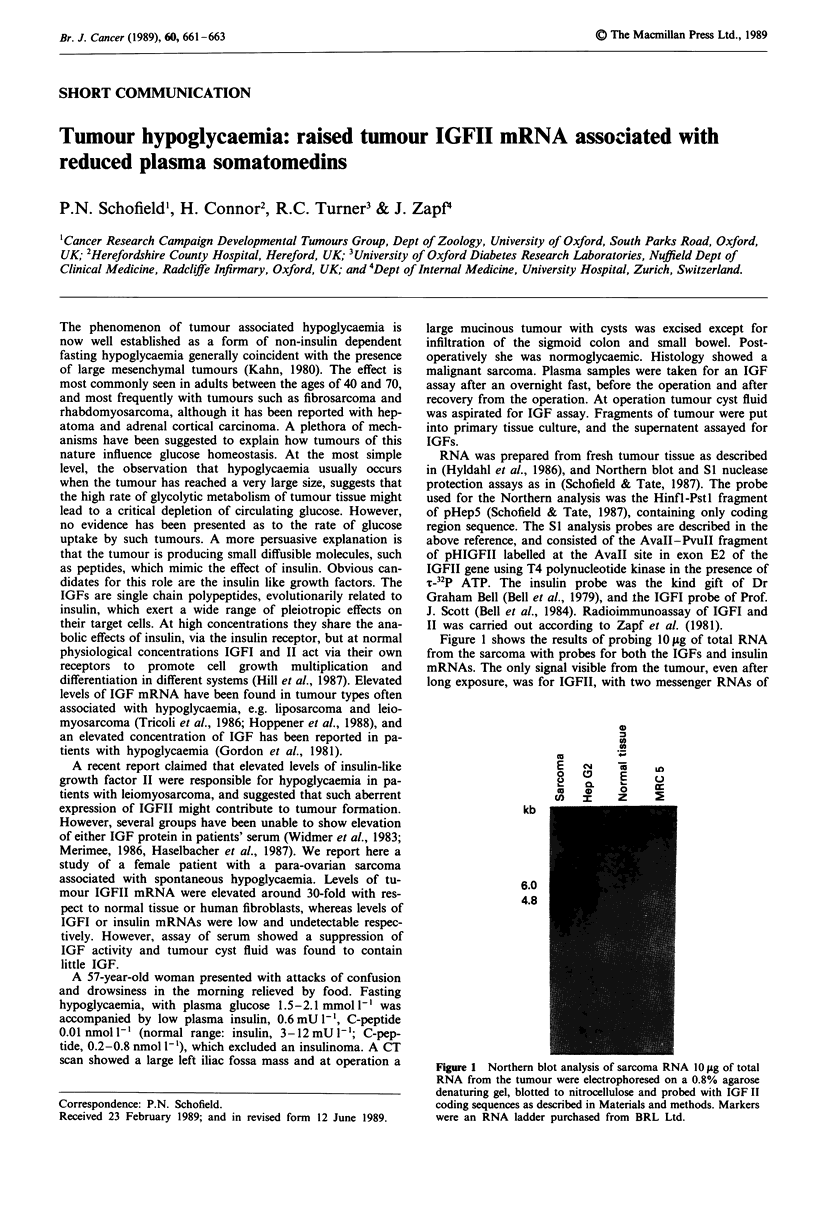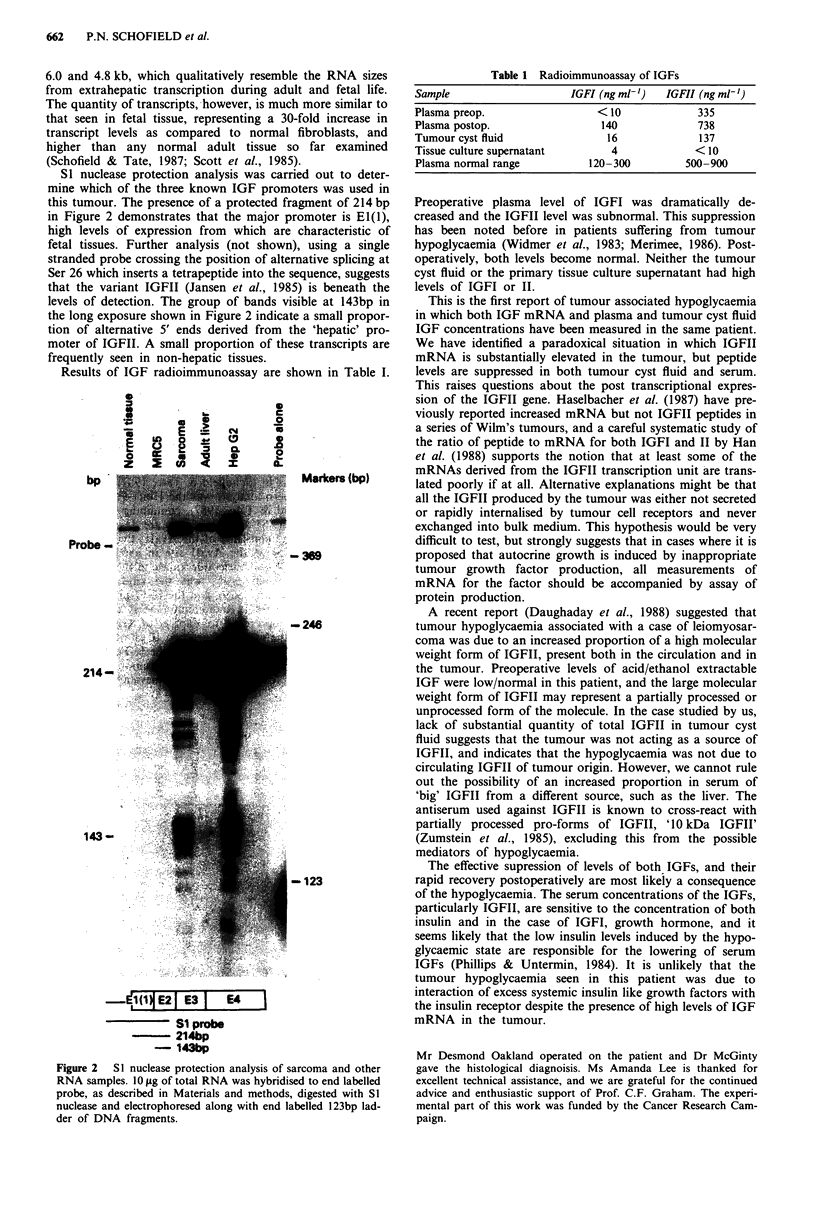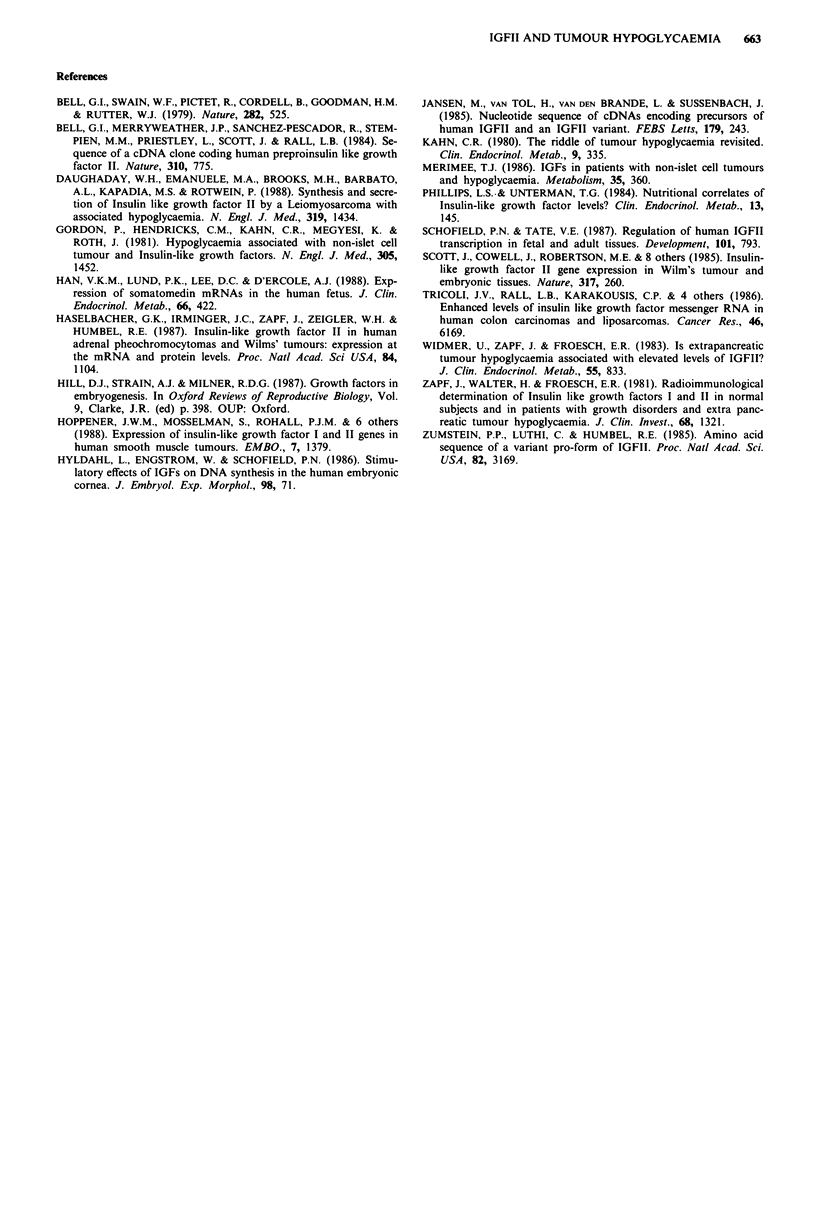# Tumour hypoglycaemia: raised tumour IGFII mRNA associated with reduced plasma somatomedins.

**DOI:** 10.1038/bjc.1989.335

**Published:** 1989-11

**Authors:** P. N. Schofield, H. Connor, R. C. Turner, J. Zapf

**Affiliations:** Dept of Zoology, University of Oxford, UK.

## Abstract

**Images:**


*Br. J. Cancer* (1989), **60**, 661–663 © The Macmillan Press Ltd., 1989

SHORT COMMUNICATION

### Tumour hypoglycaemia: raised tumour IGFII mRNA associated with reduced plasma somatomedins

P.N. Schofield[1], H. Connor[2], R.C. Turner[3] & J. Zapf[4]

[1]*Cancer Research Campaign Developmental Tumours Group, Dept of Zoology, University of Oxford, South Parks Road, Oxford, UK;* [2]*Herefordshire County Hospital, Hereford, UK;* [3]*University of Oxford Diabetes Research Laboratories, Nuffield Dept of Clinical Medicine, Radcliffe Infirmary, Oxford, UK; and* [4]*Dept of Internal Medicine, University Hospital, Zurich, Switzerland.*

The phenomenon of tumour associated hypoglycaemia is now well established as a form of non-insulin dependent fasting hypoglycaemia generally coincident with the presence of large mesenchymal tumours (Kahn, 1980). The effect is most commonly seen in adults between the ages of 40 and 70, and most frequently with tumours such as fibrosarcoma and rhabdomyosarcoma, although it has been reported with hepatoma and adrenal cortical carcinoma. A plethora of mechanisms have been suggested to explain how tumours of this nature influence glucose homeostasis. At the most simple level, the observation that hypoglycaemia usually occurs when the tumour has reached a very large size, suggests that the high rate of glycolytic metabolism of tumour tissue might lead to a critical depletion of circulating glucose. However, no evidence has been presented as to the rate of glucose uptake by such tumours. A more persuasive explanation is that the tumour is producing small diffusible molecules, such as peptides, which mimic the effect of insulin. Obvious candidates for this role are the insulin like growth factors. The IGFs are single chain polypeptides, evolutionarily related to insulin, which exert a wide range of pleiotropic effects on their target cells. At high concentrations they share the anabolic effects of insulin, via the insulin receptor, but at normal physiological concentrations IGFI and II act via their own receptors to promote cell growth multiplication and differentiation in different systems (Hill *et al.*, 1987). Elevated levels of IGF mRNA have been found in tumour types often associated with hypoglycaemia, e.g. liposarcoma and leiomyosarcoma (Tricoli *et al.*, 1986; Hoppener *et al.*, 1988), and an elevated concentration of IGF has been reported in patients with hypoglycaemia (Gordon *et al.*, 1981).

A recent report claimed that elevated levels of insulin-like growth factor II were responsible for hypoglycaemia in patients with leiomyosarcoma, and suggested that such aberrent expression of IGFII might contribute to tumour formation. However, several groups have been unable to show elevation of either IGF protein in patients' serum (Widmer *et al.*, 1983; Merimee, 1986, Haselbacher *et al.*, 1987). We report here a study of a female patient with a para-ovarian sarcoma associated with spontaneous hypoglycaemia. Levels of tumour IGFII mRNA were elevated around 30-fold with respect to normal tissue or human fibroblasts, whereas levels of IGFI or insulin mRNAs were low and undetectable respectively. However, assay of serum showed a suppression of IGF activity and tumour cyst fluid was found to contain little IGF.

A 57-year-old woman presented with attacks of confusion and drowsiness in the morning relieved by food. Fasting hypoglycaemia, with plasma glucose 1.5–2.1 $mmol\,l^{-1}$ was accompanied by low plasma insulin, 0.6 $mU\,l^{-1}$, C-peptide 0.01 $nmol\,l^{-1}$ (normal range: insulin, 3–12 $mU\,l^{-1}$; C-peptide, 0.2–0.8 $nmol\,l^{-1}$), which excluded an insulinoma. A CT scan showed a large left iliac fossa mass and at operation a large mucinous tumour with cysts was excised except for infiltration of the sigmoid colon and small bowel. Postoperatively she was normoglycaemic. Histology showed a malignant sarcoma. Plasma samples were taken for an IGF assay after an overnight fast, before the operation and after recovery from the operation. At operation tumour cyst fluid was aspirated for IGF assay. Fragments of tumour were put into primary tissue culture, and the supernatent assayed for IGFs.

RNA was prepared from fresh tumour tissue as described in (Hyldahl *et al.*, 1986), and Northern blot and S1 nuclease protection assays as in (Schofield & Tate, 1987). The probe used for the Northern analysis was the Hinfl-Pstl fragment of pHep5 (Schofield & Tate, 1987), containing only coding region sequence. The S1 analysis probes are described in the above reference, and consisted of the AvaII–PvuII fragment of pHIGFII labelled at the AvaII site in exon E2 of the IGFII gene using T4 polynucleotide kinase in the presence of $\tau$-$^{32}$P ATP. The insulin probe was the kind gift of Dr Graham Bell (Bell *et al.*, 1979), and the IGFI probe of Prof. J. Scott (Bell *et al.*, 1984). Radioimmunoassay of IGFI and II was carried out according to Zapf *et al.* (1981).

Figure 1 shows the results of probing 10 $\mu$g of total RNA from the sarcoma with probes for both the IGFs and insulin mRNAs. The only signal visible from the tumour, even after long exposure, was for IGFII, with two messenger RNAs of

**Figure 1** Northern blot analysis of sarcoma RNA 10 $\mu$g of total RNA from the tumour were electrophoresed on a 0.8% agarose denaturing gel, blotted to nitrocellulose and probed with IGF II coding sequences as described in Materials and methods. Markers were an RNA ladder purchased from BRL Ltd.

Correspondence: P.N. Schofield.
Received 23 February 1989; and in revised form 12 June 1989.

6.0 and 4.8 kb, which qualitatively resemble the RNA sizes from extrahepatic transcription during adult and fetal life. The quantity of transcripts, however, is much more similar to that seen in fetal tissue, representing a 30-fold increase in transcript levels as compared to normal fibroblasts, and higher than any normal adult tissue so far examined (Schofield & Tate, 1987; Scott *et al.*, 1985).

S1 nuclease protection analysis was carried out to determine which of the three known IGF promoters was used in this tumour. The presence of a protected fragment of 214 bp in Figure 2 demonstrates that the major promoter is E1(1), high levels of expression from which are characteristic of fetal tissues. Further analysis (not shown), using a single stranded probe crossing the position of alternative splicing at Ser 26 which inserts a tetrapeptide into the sequence, suggests that the variant IGFII (Jansen *et al.*, 1985) is beneath the levels of detection. The group of bands visible at 143bp in the long exposure shown in Figure 2 indicate a small proportion of alternative 5′ ends derived from the 'hepatic' promoter of IGFII. A small proportion of these transcripts are frequently seen in non-hepatic tissues.

Results of IGF radioimmunoassay are shown in Table I.

**Figure 2** S1 nuclease protection analysis of sarcoma and other RNA samples. 10 μg of total RNA was hybridised to end labelled probe, as described in Materials and methods, digested with S1 nuclease and electrophoresed along with end labelled 123bp ladder of DNA fragments.

**Table 1** Radioimmunoassay of IGFs

| *Sample* | *IGFI (ng ml⁻¹)* | *IGFII (ng ml⁻¹)* |
|---|---|---|
| Plasma preop. | <10 | 335 |
| Plasma postop. | 140 | 738 |
| Tumour cyst fluid | 16 | 137 |
| Tissue culture supernatant | 4 | <10 |
| Plasma normal range | 120–300 | 500–900 |

Preoperative plasma level of IGFI was dramatically decreased and the IGFII level was subnormal. This suppression has been noted before in patients suffering from tumour hypoglycaemia (Widmer *et al.*, 1983; Merimee, 1986). Postoperatively, both levels become normal. Neither the tumour cyst fluid or the primary tissue culture supernatant had high levels of IGFI or II.

This is the first report of tumour associated hypoglycaemia in which both IGF mRNA and plasma and tumour cyst fluid IGF concentrations have been measured in the same patient. We have identified a paradoxical situation in which IGFII mRNA is substantially elevated in the tumour, but peptide levels are suppressed in both tumour cyst fluid and serum. This raises questions about the post transcriptional expression of the IGFII gene. Haselbacher *et al.* (1987) have previously reported increased mRNA but not IGFII peptides in a series of Wilm's tumours, and a careful systematic study of the ratio of peptide to mRNA for both IGFI and II by Han *et al.* (1988) supports the notion that at least some of the mRNAs derived from the IGFII transcription unit are translated poorly if at all. Alternative explanations might be that all the IGFII produced by the tumour was either not secreted or rapidly internalised by tumour cell receptors and never exchanged into bulk medium. This hypothesis would be very difficult to test, but strongly suggests that in cases where it is proposed that autocrine growth is induced by inappropriate tumour growth factor production, all measurements of mRNA for the factor should be accompanied by assay of protein production.

A recent report (Daughaday *et al.*, 1988) suggested that tumour hypoglycaemia associated with a case of leiomyosarcoma was due to an increased proportion of a high molecular weight form of IGFII, present both in the circulation and in the tumour. Preoperative levels of acid/ethanol extractable IGF were low/normal in this patient, and the large molecular weight form of IGFII may represent a partially processed or unprocessed form of the molecule. In the case studied by us, lack of substantial quantity of total IGFII in tumour cyst fluid suggests that the tumour was not acting as a source of IGFII, and indicates that the hypoglycaemia was not due to circulating IGFII of tumour origin. However, we cannot rule out the possibility of an increased proportion in serum of 'big' IGFII from a different source, such as the liver. The antiserum used against IGFII is known to cross-react with partially processed pro-forms of IGFII, '10 kDa IGFII' (Zumstein *et al.*, 1985), excluding this from the possible mediators of hypoglycaemia.

The effective supression of levels of both IGFs, and their rapid recovery postoperatively are most likely a consequence of the hypoglycaemia. The serum concentrations of the IGFs, particularly IGFII, are sensitive to the concentration of both insulin and in the case of IGFI, growth hormone, and it seems likely that the low insulin levels induced by the hypoglycaemic state are responsible for the lowering of serum IGFs (Phillips & Untermin, 1984). It is unlikely that the tumour hypoglycaemia seen in this patient was due to interaction of excess systemic insulin like growth factors with the insulin receptor despite the presence of high levels of IGF mRNA in the tumour.

Mr Desmond Oakland operated on the patient and Dr McGinty gave the histological diagnoisis. Ms Amanda Lee is thanked for excellent technical assistance, and we are grateful for the continued advice and enthusiastic support of Prof. C.F. Graham. The experimental part of this work was funded by the Cancer Research Campaign.

### References

BELL, G.I., SWAIN, W.F., PICTET, R., CORDELL, B., GOODMAN, H.M. & RUTTER, W.J. (1979). *Nature*, **282**, 525.

BELL, G.I., MERRYWEATHER, J.P., SANCHEZ-PESCADOR, R., STEMPIEN, M.M., PRIESTLEY, L., SCOTT, J. & RALL, L.B. (1984). Sequence of a cDNA clone coding human preproinsulin like growth factor II. *Nature*, **310**, 775.

DAUGHADAY, W.H., EMANUELE, M.A., BROOKS, M.H., BARBATO, A.L., KAPADIA, M.S. & ROTWEIN, P. (1988). Synthesis and secretion of Insulin like growth factor II by a Leiomyosarcoma with associated hypoglycaemia. *N. Engl. J. Med.*, **319**, 1434.

GORDON, P., HENDRICKS, C.M., KAHN, C.R., MEGYESI, K. & ROTH, J. (1981). Hypoglycaemia associated with non-islet cell tumour and Insulin-like growth factors. *N. Engl. J. Med.*, **305**, 1452.

HAN, V.K.M., LUND, P.K., LEE, D.C. & D'ERCOLE, A.J. (1988). Expression of somatomedin mRNAs in the human fetus. *J. Clin. Endocrinol. Metab.*, **66**, 422.

HASELBACHER, G.K., IRMINGER, J.C., ZAPF, J., ZEIGLER, W.H. & HUMBEL, R.E. (1987). Insulin-like growth factor II in human adrenal pheochromocytomas and Wilms' tumours: expression at the mRNA and protein levels. *Proc. Natl Acad. Sci USA*, **84**, 1104.

HILL, D.J., STRAIN, A.J. & MILNER, R.D.G. (1987). Growth factors in embryogenesis. In *Oxford Reviews of Reproductive Biology*, Vol. 9, Clarke, J.R. (ed) p. 398. OUP: Oxford.

HOPPENER, J.W.M., MOSSELMAN, S., ROHALL, P.J.M. & 6 others (1988). Expression of insulin-like growth factor I and II genes in human smooth muscle tumours. *EMBO.*, **7**, 1379.

HYLDAHL, L., ENGSTROM, W. & SCHOFIELD, P.N. (1986). Stimulatory effects of IGFs on DNA synthesis in the human embryonic cornea. *J. Embryol. Exp. Morphol.*, **98**, 71.

JANSEN, M., VAN TOL, H., VAN DEN BRANDE, L. & SUSSENBACH, J. (1985). Nucleotide sequence of cDNAs encoding precursors of human IGFII and an IGFII variant. *FEBS Letts*, **179**, 243.

KAHN, C.R. (1980). The riddle of tumour hypoglycaemia revisited. *Clin. Endocrinol. Metab.*, **9**, 335.

MERIMEE, T.J. (1986). IGFs in patients with non-islet cell tumours and hypoglycaemia. *Metabolism*, **35**, 360.

PHILLIPS, L.S. & UNTERMAN, T.G. (1984). Nutritional correlates of Insulin-like growth factor levels? *Clin. Endocrinol. Metab.*, **13**, 145.

SCHOFIELD, P.N. & TATE, V.E. (1987). Regulation of human IGFII transcription in fetal and adult tissues. *Development*, **101**, 793.

SCOTT, J., COWELL, J., ROBERTSON, M.E. & 8 others (1985). Insulin-like growth factor II gene expression in Wilm's tumour and embryonic tissues. *Nature*, **317**, 260.

TRICOLI, J.V., RALL, L.B., KARAKOUSIS, C.P. & 4 others (1986). Enhanced levels of insulin like growth factor messenger RNA in human colon carcinomas and liposarcomas. *Cancer Res.*, **46**, 6169.

WIDMER, U., ZAPF, J. & FROESCH, E.R. (1983). Is extrapancreatic tumour hypoglycaemia associated with elevated levels of IGFII? *J. Clin. Endocrinol. Metab.*, **55**, 833.

ZAPF, J., WALTER, H. & FROESCH, E.R. (1981). Radioimmunological determination of Insulin like growth factors I and II in normal subjects and in patients with growth disorders and extra pancreatic tumour hypoglycaemia. *J. Clin. Invest.*, **68**, 1321.

ZUMSTEIN, P.P., LUTHI, C. & HUMBEL, R.E. (1985). Amino acid sequence of a variant pro-form of IGFII. *Proc. Natl Acad. Sci. USA*, **82**, 3169.